# A needs assessment for postgraduate training in selected public health disciplines: evidence from health services organisations in Lusaka, Zambia

**DOI:** 10.1186/s12913-020-05935-7

**Published:** 2020-11-25

**Authors:** Maio Bulawayo, Adam Silumbwe, Margarate Nzala Munakampe, Nawa Mukumbuta, Juliet Musabula, Mwimba Chewe, Chris Mweemba, Charles Michelo, Peter Hangoma

**Affiliations:** 1 Department of Health Policy and Management, School of Public Health, University of Zambia, P.O Box 50110, Lusaka, Zambia; 2 Strategic Centre for Health Systems Metrics and Evaluations (SCHEME), School of Public Health, University of Zambia, P.O Box 50110, Lusaka, Zambia; 3 Department of Epidemiology and Biostatistics, School of Public Health, University of Zambia, P.O Box 50110, Lusaka, Zambia

**Keywords:** Health policy & systems, Health services management & planning, Health economics

## Abstract

**Background:**

As most low and middle-income countries seek to achieve universal health coverage targets, there is an ever-increasing need to train human resources with the required core skills and competencies. This study reports on a needs assessment conducted among health services organisations (HSOs) to understand postgraduate training needs and service gaps for selected public health disciplines – Health Policy and Systems, Health Economics, and Healthcare Management and Planning – at the University of Zambia.

**Methods:**

The study adopted a cross-sectional design, comprising qualitative and quantitative components. Data were collected using semi-structured questionnaires administered to 32 representatives of purposively sampled public and private health service organisations based in Lusaka Zambia. The health services organisations included regulatory authorities, research institutions, government ministries, insurance firms and other cooperating partners.

**Results:**

Overall (*n* = 22), more than 68% of the stakeholders reported that they had no employees that were formally trained in the three disciplines. More than 90% of the stakeholders opined that training in these disciplines would be beneficial in providing competencies to strengthen service provision. The horizontal skills mismatch for health economics, and health services management and planning were found to be 93 and 100%, respectively. Among the critical public health training needs were: policy development and analysis, economic evaluation, and strategic management.

**Conclusions:**

This study confirms that introducing post-graduate training in the proposed public health disciplines will not only benefit Zambian health services organisations but also help strengthen the health systems in general. For other empirical contexts, the findings imply the need for the introduction of academic programmes which respond to ever-changing public health skills demanded. They should be matched with local priorities and service delivery.

**Supplementary Information:**

The online version contains supplementary material available at 10.1186/s12913-020-05935-7.

## Background

Human resource for health is one of the six building blocks of a health system. Inadequately trained or unequally distributed health workers hamper the goal of providing access to quality health care to achieve Universal Health Coverage (UHC) as espoused by the Sustainable Development Goals (SDGs). Although the gap in human resources for health for clinical areas, e.g., medicine, nursing, pharmacy, etc., has been appreciated [1, 2], the need to train complementary public health experts, such as those involved in health promotion, health services management, health policy analysis, health economics, and health systems research, among others, has received inadequate attention. As a matter of concern, and partly resulting from a limited number of trained professional, several clinicians end up taking health systems management positions and yet they are rarely equipped with the ability to manage a diverse workforce; effectively use data in decision-making, and efficiently use scarce health sector resources [3].

The limited attention on public health training in most Low- and middle-income countries (LMICs) is symptomatic of health systems that emphasize treatment and less investment in prevention. While noteworthy reductions in the burden of disease and gains in global life expectancy have been achieved over the past several decades, studies show that more could have been achieved if there was a greater emphasis on public health, or prevention, rather than treatment [4]. For example, it has been found that 60% of all premature deaths in the United States are due to factors that need public health programs, namely, behavioural, social, and environmental circumstances, while medical care can only prevent 10% of all premature deaths [5].

Developing countries are faced with similar and complex health issues that are responsible for untold levels of mortality and morbidity [6, 7], which can significantly benefit from public health approaches. For example, Cutler and Miller [8] found that water and sanitation improvement was responsible for nearly half of reduction in mortality and that this has implications for life expectancy improvements in LMICs. Other studies show that medical care may have also accounted for half the gains in global life expectancy [9]. Yet reaping full benefits of public health interventions requires appropriately trained public health professionals.

Medical care may not have the required impact if there are inefficiencies in the use of resources, for example, due to limited management capacity, inability to plan, cost, implement, and evaluate health programs [10–12]. Some of these challenges could be addressed by improving training in public health areas that include health policy, health services management, health economics, and health systems strengthening [13, 14]. Indeed, it goes without mentioning that the current double burden of communicable and non-communicable diseases grappling most LMICs requires reorganisation and realignment of health systems, which can greatly benefit from the mentioned disciplines.

In Zambia, the need to expand public health training has seen the establishment of the first-ever School of Public Health in Zambia at the University of Zambia – the leading public university in the country [15]. The School intends to introduce several postgraduate and undergraduate programmes in various fields of public health. Before introducing new training programmes, it is crucial to conduct a needs assessment to match public health training and service delivery needs, and more so, to gauge demand for the proposed training programmes [16]. Various studies have conducted training needs across different public health domains [17–20]. These studies have been very critical in the development of appropriate public health training programmes and core competencies. However, there is limited evidence, particularly in sub-Saharan Africa, around needs assessments for public health training programmes. Thus, we conducted a needs assessment among health services organisations (HSOs) to understand postgraduate training needs and service gaps for selected public health disciplines; Health Policy and Systems, Health Economics, and Healthcare Management and Planning.

## Methods

### Study design

The study adopted a cross-sectional design, comprising qualitative and quantitative components.

### Sample and sampling techniques

Thirty-two (32) respondents, each representing a purposively sampled health service organisation, were administered with semi-structured questionnaires. The sampled health service organisations included both public and private; health insurance companies, relevant government departments, public health research organisations, health professional association leaders, regulatory authorities, research institutes, and local and international organisations working in the health sector. The respondents were almost evenly split between public and private HSOs. Seventeen (17) were public HSOs, while fifteen (15) were private. Stakeholder mapping was used to identify the health services organisations as it is an incredibly useful tool when the aim is to produce a prioritised list of stakeholders for a given undertaking [21].

### Data collection

This study relied on data collected using a semi-structured questionnaire, between September and October 2018 (see attached supplementary file). Qualitative open-text data were collected using open-ended questions, while closed-ended questions were used to collect quantitative data. All the 32 respondents responded to the open-ended questions, allowing us to capture information based on complete knowledge, feeling and understanding. The participants were provided sufficient room to describe their responses in the questionnaire. The respondents were asked questions relating to their understanding of health economics, health services management and planning, and health policy and systems, and whether any people in their organisations were trained in the above disciplines. They were also asked to comment on whether there was a role in their organisations for graduates of the three disciplines, and what the specific skills these graduates should possess. Also, they were asked questions on whether training in the three disciplines was beneficial to their organisations. The questions, developed after an extensive review of similar literature were piloted and refined before the commencement of data collection. The data collection was conducted by trained researchers (some of whom are co-authors) with a good understanding of the study disciplines as well as research practice.

### Data analysis

The data collected were entered, organised and analysed in Microsoft Excel 2013. The analysis of quantitative data was conducted in the following manner. Firstly, we analysed the proportion of organisations who reported to have had people formally trained and the level of training in the three public health disciplines: Health Policy and Systems, Health Economics, and Healthcare Services Management and Planning. Secondly, we examined the skill gaps by examining the proportion of organisations who would find training across the three disciplines beneficial. In particular, we present the proportion of respondents who thought that training in each of the three disciplines would be beneficial to their organisations as well as their rating of the skills gap for each discipline. Relatedly, for each discipline, the specific skills required in order of importance were also identified.

Lastly, we assessed the skills mismatches relating to health economics and health services management and planning. The concept of ‘skill mismatch’ is multidimensional. It is commonly defined in two ways: vertical or horizontal mismatch. Vertical skills mismatch refers to a situation where the level of education or skills is either less or more than is required to perform a given job. On the other hand, horizontal skills mismatch exists whenever an employee’s level of education or skills is not appropriate to perform a given job [22]. In this study, we defined the skills mismatch from a horizontal perspective. In particular, a skills mismatch is said to exist whenever we have employees performing roles relating to Health Policy and Systems, Health Economics, and Healthcare Management and Planning, for which they are not formally trained.

The qualitative open text data were analysed using thematic analysis [23]. Since the data was in open text format, it was considered to be already transcribed by the research team. Thus, all the text information was iteratively read among the research team members to identify emergent themes, while the structural themes were organised according to the three public health disciplines; health policy and systems, health economics, and health services management and planning. The notes that were captured during the data collection were also used to inform emergent themes in each of the three disciplines. Some of the emergent themes centred around identification of critical competencies and potential functions of people that would be trained. Most of the perspectives on the need for training in the selected public health disciplines were similar across all thematic areas.

### Ethical considerations

This study was granted ethical exemption by the University of Zambia Biomedical Research Ethics Committee (UNZABREC) as it was deemed to fall under non-human subject research. Administrative permission to conduct the study was obtained from the National Health Research Authority (NHRA) as provided by the Zambian law. We ensured confidentiality by de-identifying all the data, and only respondents who verbally consented were interviewed.

## Results

All the 32 study participants (Table 1) who were identified during the stakeholder mapping responded to the questionnaire, providing both detailed open-text and closed-ended responses, representing a 100% response rate. The open-text responses are presented as verbatim quotes using the exact text as provided by the respondents. We report on the levels of training in the specified public health disciplines, as well as the participant perspectives on the skills gaps, training needs and skills-mismatch among the sampled health services organisations.

**Table 1:** Participant Categories

Category	Organisation	# of Interviews
1. Regulatory authorities	Zambia National Medical Regulatory Authority (ZAMRA)	2
	General Nursing Council of Zambia (GNCZ)	2
	Zambia Medical Association (ZMA)	2
	Health Professional Council of Zambia (HPCZ)	2
	National Aids Council	1
2. Line Ministries and government departments	Ministry of Health- Directorate Public Health, Dept of Policy and Planning	1
	Ministry of National Development Planning	1
	Zambia Correctional Services	1
3. Health Insurance	SANCARE Insurance (private)	2
	Prudential Insurance (private)	2
4. Health services	Zambia Medical Stores Ltd.	2
	Churches Health Association of Zambia	2
	ST Johns Private Hospital	2
	Right to Care EQUIP	1
	Right to care Zambia	1
5. International Partners	World Bank Country Office	1
	World Health Organisation Country Office	2
6. Public health Research	Population Council–Zambia	2
	Akros Research	2
	British American Tobacco	1
Total Participants		32

### Level of training in selected public health disciplines in the organisations

The respondents indicated whether their organisation had anyone with some training in each of the three public health disciplines (see Table 2). For health policy and systems, 56% of the respondents indicated that no one had been formally trained in their organisation. For those who reported having had some form of training in the health policy and systems, 19% were trained at masters’ level, 13% had received in-service training, 9% had been trained at the bachelor’s degree level, and 3% at the PhD level. However, it is essential to note that in almost all instances, the health policy and systems training was received as a part of training in other public health disciplines and not as a specialist field of study.

**Table 2:** Level of Training across the Three Disciplines

Discipline	Level of Training							Total
	No Training	Trained						
		In-service	Certificate	Diploma	Degree	Masters	PhD	
**Health Policy and Systems**	18 (56%)	4 (13%)	0 (0%)	0 (0%)	3 (9%)	6 (19%)	1 (3%)	32 (100%)
**Health Economics**	21 (66%)	1 (3%)	0 (0%)	0 (0%)	1 (3%)	9 (28%)	0 (0%)	32 (100%)
**Health Services Mgt. & Planning**	25 (78%)	1 (3%)	0 (0%)	0 (0%)	1 (3%)	5 (16%)	0 (0%)	32 (100%)

Only 34% of the respondents reported having someone with some training in health economics in their organisation. Among those trained, 3% received in-service training, 3% were trained at the bachelor’s degree level, and 28% at the master’s degree level. In terms of full or specialized training, only one respondent reported having had someone in the organisation specially trained in health economics; at the master’s level. The rest received health economics training either in-service or as part of other public health related training.

For health services management and planning training, only 22% of the respondents reported having had some training in health services management and planning. Of these, 3% had received in-service training, 3% at the bachelor’s degree level, and 16% at the master’s degree level. Strikingly, none of the people trained had full or specialized training in health services management and planning.

### Importance of training in the selected public health disciplines

Almost all organizations indicated that training in the proposed public health disciplines would be beneficial to their organizations (see Table 3). We found that 94% of the respondents felt that specialized postgraduate programs in health policy and systems, as well as health economics, would be very beneficial to their organisations. Also, 91% of the respondents were of the view that formal training in health services management and planning would benefit their organisations.

**Table 3:** Whether the Training Would be Beneficial to the Organisations

Discipline	Yes	No	Do Not Know	Total
**Health Policy and Systems**	30 (94%)	0 (0%)	2 (6%)	32 (100%)
**Health Economics**	30 (94%)	2 (6%)	0 (0%)	32 (100%)
**Health Services Mgt. & Planning**	29 (91%)	3 (9%)	0 (0%)	32 (100%)

In terms of the extent of benefit, we asked respondents to indicate, on a Likert scale (High, Medium, and Low), how important specialized training for each of the specified disciplines would be for their organisation. Most respondents indicated that the need is high (see Table 4). In particular, across all three disciplines, at least two-thirds of the respondents were of the view that formal training in these fields was a matter of agency. In contrast, at least a quarter of the respondents thought that the skills gap across the three fields was moderate.

**Table 4:** Ranking of the Importance of Training in Each Programme

Discipline	High	Medium	Low	Total
**Health Policy and Systems**	23 (72%)	8 (25%)	1 (3%)	32 (100%)
**Health Economics**	22 (69%)	9 (28%)	1 (3%)	32 (100%)
**Health Services Mgt. & Planning**	21 (66%)	8 (25%)	3 (9%)	32 (100%)

### Perspectives on the skills training needs in the selected public health disciplines

The study participants provided detailed descriptions of the reasons why training in the three selected public health disciplines was vital, and more so, what kind of competencies they expected to acquire and how they would benefit practice in their organisations. Furthermore, they described the potential contribution of personnel that would be trained in the selected disciplines to the health system in general.

#### Health policy and systems training

Most of the participants reported that having many professionals trained in health policy and systems would not only provide competencies to improve the running of their organisations but the health sector at large. They indicated building such capacity would contribute to a paradigm shift, from solely focusing on clinical functions within the health system to acknowledging the role of supporting functions and how they interact with broader structural factors in the provision of health services. Furthermore, health policy and systems training was said to be critical in providing skills such as change management, as well as being able to set and achieve strategic goals for population health improvement within health systems. It was also stated that health policy and systems training at postgraduate level had a more significant role to play with regards to facilitating innovative research to strengthen health systems through providing evidence to navigate some of the bottlenecks of the system in the provision of essential health services.

*“Zambia has a gap in this health policy and systems training and if this is strengthened, it means even employees in Ministry of Health will be keen to carry out the right procedures when it comes to health systems strengthening. These skills will benefit both the private and public health sectors.” [KII 23, Research]*

*“Very essential especially for Directors because they are the policy makers. They need to know what it takes and the impact of their decisions on health services. They also need to be able to do research.” [K1I15, Government Ministry]*

*“Trained people would know what to do and how to go about formulating policies. When evidence is provided trained personnel would know how to transform it into policy brief and present it to the government.” [KII19, Cooperating partner]*

#### Health economics training

For health economics, the respondents reported that it would benefit their organisations by providing competencies to facilitate efficient use of meagre health resources. Priority setting was widely mentioned as one of the critical competencies students trained in health economics would have to acquire. Health economics was reported to be vital in building capacity to formulate, evaluate health policies and strategies using economics and econometric approaches. Furthermore, this training would help the health sector personnel to participate adequately in shaping health policy at both the formulation and implementation stages. The participants indicated that health policy implementation remained a challenge in Zambia, and building such capacity would go a long way in improving policy outcomes. It was also reported that introducing health economics training at the University of Zambia could enable the Zambian health sector to count on a pool of locally trained Health Economists that have a full understanding of the local contexts as opposed to outsourcing from outside the country, which is the practice in most organisations. Some of the organisations indicated that they outsourced for activities such as economic evaluations, and introducing health economics training would help address this gap and reduce costs.

*“This would be important for our organisation to conduct research, analyse policy and participate in policy shaping and development of health financing” [KII18, Regulatory]*

*“This training would benefit the organisation because many health service providers we deal with lack the necessary acumen to efficiently manage their health services delivery. This often leads to disputed insurance claims.” [KII 27, Insurance]*

#### Healthcare services management and planning training

The participants reported that training people in management skills such as planning and strategic management would greatly benefit their institutions as well as the health system. They stated that management training contributed to the efficiency with which health services were provided to society. For example, they indicated that the Ministry of Health (MoH) would benefit from personnel trained in the planning of health services at various levels. The participants not only stated the importance of management training at post-graduate level but also highlighted the need for undergraduate training. It was suggested that personnel with undergraduate training would execute operational level management functions within the health system, whilst higher level management decision making and research would be the focus of the postgraduate training. Furthermore, management training was said to be vital in proving competencies to enhance public health leadership across health services teams and organisations.

*“We will be more precise in planning and avoid budget variations. Secondly, it would also be more beneficial to planners in MoH. Currently they employ people who have done development studies. But the demographers don't have a good understanding of the health system” [K14, Regulatory]*

*“Effective health services management and planning will help assist both public and private institutions in planning and managing resources and programs effectively in organisations where resources are finite.” [K22, Health services]*

#### Skills training needs

We also identified the particular skills training needed in order of importance and relative frequency of responses on the need for selected skills. These are summarized in Table 5. For health policy and systems training, the top skills desired were policy analysis and planning (72%), monitoring and evaluation of health programs (72%), and health systems research capacity (72%); followed by implementation and management of health programs (56%), change management (53%), and training relating to policy, politics and power (32%). For health economics training, the most critical skills needs are health care financing (75%), economic evaluation of health programs (75%); followed by decision-analytic modelling (59%), and health economics research capacity (59%). There is also a demand for training in the operations of the health insurance market (56%) and measuring health system efficiency (53%). For health services management and practice, the most sought out skill was strategic management in health programs (69%), followed by leadership and management in health programs (66%), and program implementation (56%).

**Table 5:** Competency Needs by Discipline

Health Policy and Systems	Health Economics	Health Services Management and Planning
Policy analysis and planning (72%) Monitoring and evaluating health programmes (72%) Health systems research capacity (72%) Implementing and managing programmes in Health systems (56%) Managing change in health systems (53%) Policy, politics and power (34%)	Health care financing (75%) Economic evaluation of health programmes (75%) Decision analytic modelling (59%) Health economics research capacity (59%) Understanding the health insurance markets (56%) Measuring health system efficiency (53%)	Strategic management in Health programmes (69%) Leadership and management in Health programmes (66%) Implementing programmes in the health system (56%)

### Skills mismatch

An alternative way of looking at the skills gap is to look at the skills mismatch – the misplacement of skills for a given job description. In this study, this is taken to imply a situation where a person not formally trained (as part of a specialist programme) to perform roles related to a given public health field is performing those roles. We found significant horizontal skills mismatch in both health economics and health services management and planning (see Table 6). All people performing functions relating to health services management and planning were not formally trained to accomplish them. While some of the respondents performed roles of strategic management and leadership in health programmes, none of them was officially trained in these competencies. For health economics roles relating to health care financing, evaluation of health programmes and the assessment of health system efficiency, 93% of people performing them were not formally trained.

**Table 6:** Level of Skills Mismatch

Disciplines	Someone Performing Related Roles	Formally Trained for the Role	Skills Match	Skills Mismatch
**Health Economics**	14	1	79%	93%
**Health Services Mgt. & Planning**	14	0	0%	100%

## Discussion

This study has highlighted the significant skill gaps which exist across selected public health disciplines in Zambian health services organisations and the need to introduce formal training programmes. The identified skill gaps are not unique to Zambia. Still, they have also been reported in other Low- and middle-income countries (LMICs). This needs assessment provides vital evidence to inform the development of curriculum content that is relevant to industry and practice. Furthermore, it provides a picture of priority competencies expected from the potential graduates of the selected public health programmes, as well as the likely challenges that these programmes may face both from within the university system and external environment.

Gilson et al. underscore the potential contribution of health policy and systems training to societal and national development efforts [24]. However, training capacity is inadequate in most settings, including Zambia. Uzochukwu et al. report on the inadequacy in health policy and systems capacity in Nigeria, and the subsequent reliance on the high-income countries for training [25], which has equally been the case for Zambia. This reliance stems partly from poor funding of LMIC schools of public health [26]. The persistency of limited funding and reliance on high-income countries hinders the development of locally driven public health solutions. In this regard, the study participants indicated that those trained in health policy and systems should possess such competencies as policy analysis, change management, policy formulation and implementation, as well as the capacity to navigate political influences in the health system.

The skills gaps in health economics, health care financing and economic evaluation that we found in Zambia have also been highlighted in a recent study on health economics knowledge needs assessment in Latin America [27], and many other LMICs [28, 29]. The severe healthcare financing challenges in LMICs, where health budgets are tight, yet the burden of disease is high, suggest that the need for health economics skills is dire to ensure adequate prioritisation of health technologies [29]. There have been discussions of south-south collaboration, which is the exchange of resources, technology, and knowledge between developing countries of the Global South, to leverage skills in health economics and health technology assessment. Part of the reason is that these countries share similar problems and can benefit from shared solutions. The biggest challenge is that even this south-south collaboration requires financial resources which ultimately requires assistance from high income countries. Nonetheless, strong human resource capacity and training in health economics and healthcare financing may produce a cadre that may come up with innovative financing solutions and reduce resource waste through efficiency gains.

The biggest gap in public health training identified in this study is in health services management and planning. Appropriate management is key to ensuring that human resources, medical resources and supplies, infrastructure, and financial resources are carefully organised to produce the highest possible health gain. The depth of the skills gap in health services management and planning is also pronounced in other sub-Saharan African contexts. For example, a self- assessment of relevant health services management skills among healthcare managers in South Africa found significant skills gaps in strategic planning, health delivery and people management [30]. Management training in public health has lagged in Zambia like other LMICs. Still, it remains fundamental to health system governance strengthening efforts. Thus, this training provides an opportunity for the development of potentially effective approaches to the management of our local health systems. The training may also provide a platform for the development of leadership interventions that can nurture new forms of leadership that respond to contemporary public health challenges [31].

Apart from adequate resources, two other factors may hinder the establishment and successful execution of the proposed training across the identified public health disciplines. First is the regulatory bureaucracy in healthcare, and particularly in the approval of academic programmes in most LMIC [26]. At the University of Zambia (UNZA), a very centralised management system requires those with ideas such as introducing a new programme to go through various committees, and the approval process can take years. After this needs assessment and several years of making attempts, regulatory approval has been granted to UNZA for the introduction of postgraduate training in health economics, health policy and management, and health services management and planning for the 2020/21 academic year. However, resource challenges such as inadequate teaching facilities and human resources remain.

Secondly, political will has long been recognised as crucial to the implementation of public health programmes [32]. There is need for commitment and recognition from MoH that training beyond clinical areas is crucial so that these training needs are included in health sector training strategic plans. This political will has to translate to action, where budgets are allocated for supporting training. In Zambia, and many other LMIC, there are education financing schemes for in-service for clinical areas, but few or none for specific public health programs. This lack leads to subdued demand among financially challenged students. However, the Zambia MoH and UNZA have had a very mutual collaboration in public health research. For example, UNZA periodically supports the Ministry in monitoring and evaluating progress and performance for many of the latter’s strategic plans, including successive National Health Strategic Plans. Therefore, there is potential to leverage on this long-standing engagement to attract funding for the three newly approved academic programmes.

The current global health challenges call for the schools of public health in Zambia and other LMICs to reposition themselves to train relevant cadres that respond adequately to existing and emergent issues affecting local health systems. Enhancing the quality of graduates as well as their key competencies will require reviewing traditional approaches to health professional training through collaboration between academic institutions and key industry players. Whilst this study contributes to efforts of developing a curriculum relevant to local contextual needs, more and consistent engagement with health services organisations is required to keep abreast with some of the public health challenges and training requirements.

### Strengths and limitations

The open text data was collected from a varied sample which allowed for triangulation of views from the many sources [33, 34]. It also allowed the capture of training needs in words of the participants adding validity to the findings [35]. Also, the data analysis was iteratively done by the research team, adding to the credibility of the research processes [33].

The study has provided substantial evidence on the skill gaps in critical public health disciplines that could apply to other sub-Saharan Africa countries. This study makes an essential contribution to literature as there are limited studies that have assessed skills needs in the selected public health disciplines. Furthermore, we believe that our design is comparable or improves upon previous needs assessments, most of which are a mostly informal collection of ideas, and not systematic assessments.

However, the study has several limitations. Firstly, the study was ‘static’ in nature in the sense that it was unable to track the changing skills needs in a dynamic healthcare sector. Secondly, the study took a demand-side perspective without incorporating the supply-side. Thirdly, the qualitative component of this study was limited to open text, and not actual discussions with the respondents, which defined the depth of qualitative data that was collected. Lastly, the few health services organisations in Zambia limited the number of study participants; thus, individuals representing organisations were sampled.

## Conclusions

Inadequate and/or poorly trained human resources for health remains a significant constraint to achieving Universal Health Coverage – a key policy objective of many countries around the world. This study assessed the skill gaps and mismatches in health policy and systems, health services management and planning, and health economics in the Zambian health sector. The study found significant skill gaps across all the three disciplines and significant skill mismatches were identified in health economics, and health services management and planning.

We conclude that the goal of achieving Universal Health Coverage requires going beyond the focus on clinical training, and supporting the introduction of structured training programmes in the three public health disciplines. Nonetheless, we recommend a continuous assessment of public health training needs, given the ever-changing training needs of the health sector. Such reviews will help academics to tailor public health training to local context needs.

## Supplementary Information

**Additional file 1.**

## Data Availability

Key data summaries are included in the manuscript. The full dataset used for the current study are available from the corresponding author on request.

## Abbreviations

HSOs: Health Service Organisations
LMICs: Low- and Middle-Income Countries
MoH: Ministry of Health
NHRA: National Health Research Authority
SDGs: Sustainable Development Goals
UHC: Universal Health Coverage
UNZABREC: University of Zambia Bioethics Research Committee
