# Supplementary material for: A needs assessment for postgraduate training in selected public health disciplines: evidence from health services organisations in Lusaka, Zambia
Source: BMC Health Serv Res. 2020 Nov 25;20:1079. doi: 10.1186/s12913-020-05935-7 (PMC7689991; doi:10.1186/s12913-020-05935-7)
Supplement: Supplementary file 1 — Additional file 1. [file 12913_2020_5935_MOESM1_ESM.pdf]

My name is……… from the University of Zambia. I am a member of staff in the Department of Health Policy and Management, School of Public Health. We are grateful that you have spared some time to have this discussion with us. The aim of this interview is to identify health systems training needs and service gaps for selected public health disciplines (Health Policy and Systems, Health Economics, and Health Services Management and Planning).

MAIN QUESTION	Probe	RESPONSE/ COMMENT
**BACKGROUND INFORMATION**		
1. Kindly tell me about yourself?	What do you do as part of your work at this institution/organization?	a) Position<br>• Managerial…………………………………………………..<br>• Technical……………………………………………………<br>• Clerical………………………………………………………<br><br>b) Department:<br>• Administration/Support services……………………………<br>• Clinical/Health services…………………………………….<br>• Research/Public Health……………………………………..<br><br>c) Health Service Organisation:<br>• Public HSO (specify)……………………………………..<br>• Private HSO(Specify)……………………………………..
**HEALTH POLICY AND SYSTEMS TRAINING**		
2. What do you understand by health policy and systems?<br><br>Health Policy and systems research focuses on understanding and	a. What do you understand by health systems it/application	
	b. What do you understand by health systems it/application (health policy)	
	c. Do you have any one performing functions related health policy and systems? – Describe the roles	

improving how societies organize themselves in achieving collective health goals, and how different actors interact in the policy and implementation processes to contribute to policy outcomes.		
	**d.** Do you know of any people in this institution that have been trained or plans to train in Health Policy, Health Systems or Health Policy and system research? **(IF no skip to Q2 f.)**	a) Yes, Health policy<br>b) Yes, heath systems<br>c) Yes, Health policy and systems research<br>d) No<br>**Can have multiple responses for 'yes'**
	**e.** If yes, at what level of training? **(Skip to Q2 h.)**	**Health Policy**<br>a) In-service training (up to 1 month)<br>b) Certificate (up to 1-year training)<br>c) Diploma (more than 1 year to 3 years)<br>d) Degree (4 years)<br>e) Masters<br>f) PhD<br>g) Other (Specify) ……………………………………………<br>**Health Systems**<br>a) In-service training (up to 1 month)<br>b) Certificate (up to 1-year training)<br>c) Diploma (more than 1 year to 3 years)<br>d) Degree (4 years)<br>e) Masters<br>f) PhD<br>g) Other (Specify)……………………………………………<br>**Health Policy and Systems Research**<br>a) In-service training (up to 1 month)<br>b) Certificate (up to 1-year training)<br>c) Diploma (more than 1 year to 3 years)<br>d) Degree (4 years)<br>e) Masters<br>f) PhD<br>g) Other (Specify) ……………………………………………

	**f.** Do you think there is any role for people with such training in your organization/institution? **(IF no or Don't Know skip to Q2 h.)**	a) Yes<br>b) No<br>c) Don't know
	**g.** If yes, what role would that be?	a) Programme Directors/Managers<br>b) Programme Officers<br>c) Researchers<br>d) Health systems strengthening<br>e) Other (Specify)...
	**h.** If this training was to be offered, what could be some of the skills you would want such a cadre to have?	a) Policy Analysis and Planning<br>b) Policy, Politics & Power<br>c) Implementing and managing programs in the Health System<br>d) Monitoring and Evaluating health programs<br>e) Health System Research Capacity<br>f) Managing Change in Health Systems<br>g) Other (Specify)...
	**i.** How would you rate the skills gap of this training in the healthcare industry –Explain?	a) Very high..<br>b) Medium...<br>c) Low..
	**j.** Do you think this training would be very beneficial for institutions like yours? **(If no or don't know skip to Q3 a.)**	a) Yes<br>b) No<br>c) Don't Know
	**k.** If yes, why? -employment prospects? – explain	
**HEALTH ECONOMICS TRAINING**		

What do you understand by the term Health Economics.<br><br>Health Economics is an applied field of study that uses economic theories of consumer, producer and social choice to understand the behaviour of individuals, health care providers, public and private organizations, and governments in decision-making Health economists apply the theories of production, efficiency, disparities, competition, and regulation to better inform the public and private sector on the most efficient, cost-	**a.** What do you understand by it/ application	
	**b.** Do you have any one performing functions related health economics? – Describe the roles	
	**c.** Do you know of any people in this institution/organization that have been trained or plans to train in Health Economics? **(IF 'No' skip to Q3 e.)**	a) Yes<br>b) No
	**d.** If yes, at what level of training? **(Skip to Q3 g.)**	a) In-service training (up to 1 month)<br>b) Certificate (up to 1 year training)<br>c) Diploma (more than 1 year to 3 years)<br>d) Degree (4 years)<br>e) Masters<br>f) PhD<br>g) Other (Specify)…………………………………………………
	**e.** Do you think there is any role for people with such training in your organization/institution? **(IF 'No' or 'Don't Know' skip to Q3 g.)**	a) Yes<br>b) No<br>c) Don't Know
	**f.** If yes, what role would that be?	a) Programme Directors/Managers<br>b) Programme Officers<br>c) Researcher<br>d) Health Economist<br>e) Other (Specify)………………………………………………..

effective and equitable course of action.<br>3.	**g.** How would you rate the skills gap of this particular training in the healthcare industry – Explain?	a) Very high……………………………………………………<br>b) Medium…………………………………………………….<br>c) Low…………………………………………………………..
	**h.** If this training was to be offered, what could be some of the skills you would want such a cadre to have?	a) Understanding of Health Care Financing<br>b) Economic Evaluation of health programs<br>c) Understanding of Health insurance market<br>d) Decision Analytic Modelling<br>e) Measuring Health System Efficiency<br>f) Health Economics Research Capacity<br>g) Other (Specify)…………………………………………………….
	**i.** Do you think this training would be very beneficial for institutions like yours? **(IF 'No' or 'Don't Know' skip to Q4 a.)**	a) Yes<br>b) No<br>c) Don't Know
	**j.** If yes, why? -employment prospects?	
**HEALTH SERVICES MANAGEMENT AND PLANNING**		
4. What do you understand by the term Health Services Management and Planning	**a.** What do you understand by it/ application	
	**b.** Do you have any one performing functions related healthcare management and planning? – Describe the roles	
	**c.** Do you know of any people in this institution/organization that have been trained or plans to train in Health Services Management and Planning? **(IF 'No' skip to Q4 f.)**	a) Yes<br>b) No

	**d.** If yes, at what level of training? **(Skip to Q4 g.)**	a) In-service training (up to 1 month)<br>b) Certificate (up to 1 year training)<br>c) Diploma (more than 1 year to 3 years)<br>d) Degree (4 years)<br>e) Masters<br>f) PhD<br>g) Other (Specify)……………………………………………..
	**e.** Do you think there is any role for people with such training in your organization/institution? **(IF 'No' or 'Don't Know' skip to Q4 g.)**	a) Yes<br>b) No<br>c) Don't know
	**f.** If yes, what role would that be?	a) Programme Directors/Managers<br>b) Programme Officers<br>c) Other (Specify) ………………………………………………..
	**g.** If this training was to be offered, what could be some of the skills you would want such a cadre to have?	a) Leadership and Management in Health programs<br>b) Implementing programs in the Health System<br>c) Monitoring and Evaluation of programs in the Health System<br>d) Strategic Management in health programs<br>e) Health Economics Research Capacity<br>f) Other (Specify)………………………………………………..
	**h.** How would you rate the skills gap of this particular training in the healthcare industry – Explain?	a) Very high……………………………………………………<br>b) Medium…………………………………………………….<br>c) Low…………………………………………………………
	**i.** Do you think this training would be very beneficial for institutions like yours?	a) Yes<br>b) No<br>c) Don't know
	**j.** If yes, why? -employment prospects? – Explain	

**Thank you for your time**		
